# Supplementary material for: Antimicrobial Lock Therapy in Clinical Practice: A Scoping Review
Source: Microorganisms. 2025 Feb 13;13(2):406. doi: 10.3390/microorganisms13020406 (PMC11857916; doi:10.3390/microorganisms13020406)
Supplement: Supplementary file 1 [file microorganisms-13-00406-s001.zip › microorganisms-3411513 Supplementary Figures.pdf]

Supplementary Material Figures S1 and S2, Images: ROB2 quality assessment

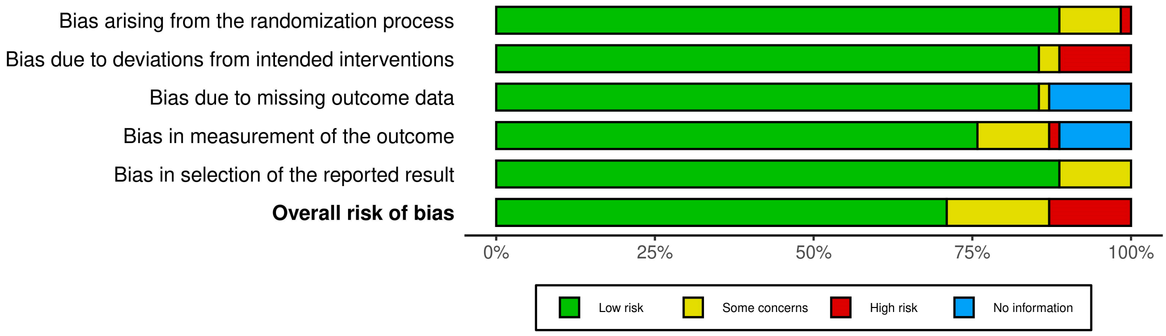

Risk of bias domains

Study	D1	D2	D3	D4	D5	Overall
(Al-Ali 2018)	+	+	+	+	+	+
(Al-Hwiesh 2007)	-	+	-	X	+	X
(Al-Hwiesh 2008)	-	+	?	+	+	-
(Allison 2014)	+	X	?	?	-	X
(Aniort 2019)	+	+	+	+	+	+
(Betjes 2004)	+	+	+	+	+	+
(Bisseling 2010)	-	+	+	-	+	-
(Bonkain 2021)	+	+	+	+	+	+
(Broom 2009)	+	+	+	+	+	+
(Broom 2012)	+	+	+	+	+	+
(Campos 2011)	-	+	+	-	+	-
(Carratalà 1999)	+	+	+	+	+	+
(Coli 2010)	+	X	?	?	-	X
(Davanipur 2011)	X	X	?	?	-	X
(Decembrino 2014)	+	X	?	?	-	X
(Dogra 2002)	+	+	+	+	+	+
(Dümichen 2012)	+	+	+	+	+	+
(Ezzat 2023)	+	+	+	-	+	-
(Feeney 2022)	+	X	?	?	-	X
(Filiopoulos 2011)	+	+	+	-	+	-
(Filippi 2007)	+	+	+	-	+	-
(Garland 2005)	+	+	+	+	+	+
(Gudiol 2018)	+	+	+	+	+	+
(Gudiol 2020)	+	+	+	+	+	+
(Handrup 2013)	+	+	+	+	+	+
(Islam 2024)	+	+	+	+	+	+
(Kanaa 2015)	+	+	+	+	+	+
(Kayton 2010)	+	+	+	+	+	+
(Khosroshahi 2015)	+	X	?	?	-	X
(Kim 2006)	-	+	+	-	+	-
(Klek 2015)	+	+	+	+	+	+
(Lesens 2024)	+	+	+	+	+	+
(Longo 2017)	+	+	+	+	+	+
(Lopes 2019)	+	+	+	+	+	+
(Luiz 2017)	+	+	+	+	+	+
(Łyszkowska 2019)	+	-	+	+	+	-
(Maki 2011)	+	+	+	+	+	+
(Moghaddas 2015)	-	+	+	-	+	-
(Moran 2012)	+	+	+	+	+	+
(Mortazavi 2011)	+	+	+	+	+	+
(Nassiri 2023)	+	+	+	+	+	+
(Nori 2006)	+	+	+	+	+	+
(Pérez-Granda 2014)	+	+	+	+	+	+
(Pérez-Granda 2020)	+	+	+	+	+	+
(Rijnders 2005)	+	+	+	+	+	+
(Rijnders 2019)	+	+	+	+	+	+
(Salonen 2018)	+	+	+	+	+	+
(Saxena 2006)	+	+	+	+	+	+
(Schoot 2015)	+	+	+	+	+	+
(Slobbe 2010)	+	+	+	+	+	+
(Sofroniadou 2012)	+	+	+	+	+	+
(Sofroniadou 2017)	+	+	+	+	+	+
(Solomon 2010)	+	+	+	+	+	+
(Souweine 2015)	+	+	+	+	+	+
(Tribler 2017)	+	+	+	+	+	+
(van den Bosch 2024)	+	+	+	+	+	+
(Wang 2012)	+	X	?	?	-	X
(Wolf 2017)	+	+	+	+	+	+
(Wolf 2018)	+	+	+	+	+	+
(Worth 2014)	+	+	+	+	+	+
(Wouters 2018)	+	+	+	+	+	+
(Zhang 2009)	+	-	+	+	+	-

Domains:
D1: Bias arising from the randomization process.
D2: Bias due to deviations from intended intervention.
D3: Bias due to missing outcome data.
D4: Bias in measurement of the outcome.
D5: Bias in selection of the reported result.

Judgement
X High
- Some concerns
+ Low
? No information

Supplementary Material Figures S3 and S4, Images: ROBINS-I quality assessment

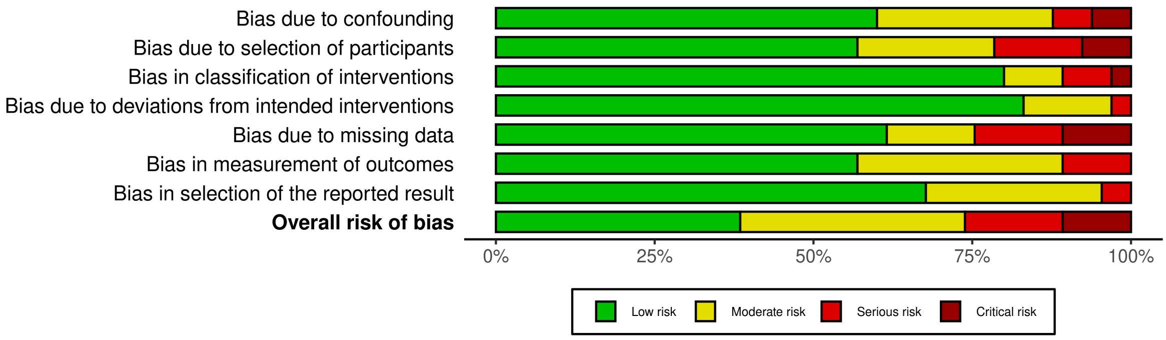

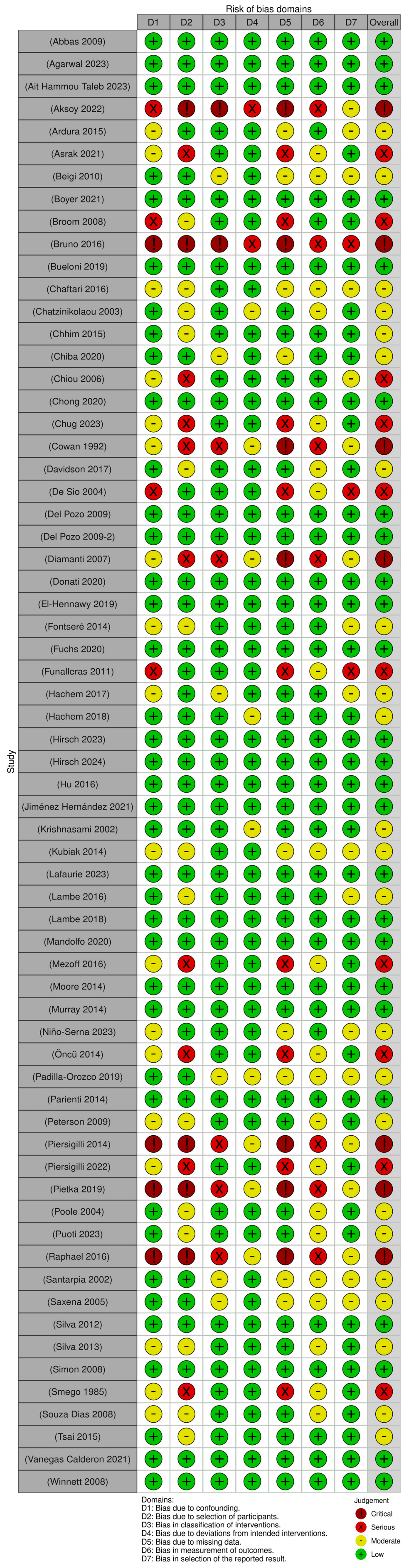
Risk of bias domains
D1
D2
D3
D4
D5
D6
D7
Overall
Study
(Abbas 2009)
(Agarwal 2023)
(Ait Hammou Taleb 2023)
(Aksoy 2022)
(Ardura 2015)
(Asrak 2021)
(Beigi 2010)
(Boyer 2021)
(Broom 2008)
(Bruno 2016)
(Bueloni 2019)
(Chaftari 2016)
(Chatzinikolaou 2003)
(Chhim 2015)
(Chiba 2020)
(Chiou 2006)
(Chong 2020)
(Chug 2023)
(Cowan 1992)
(Davidson 2017)
(De Sio 2004)
(Del Pozo 2009)
(Del Pozo 2009-2)
(Diamanti 2007)
(Donati 2020)
(El-Hennawy 2019)
(Fontseré 2014)
(Fuchs 2020)
(Funalleras 2011)
(Hachem 2017)
(Hachem 2018)
(Hirsch 2023)
(Hirsch 2024)
(Hu 2016)
(Jiménez Hernández 2021)
(Krishnasami 2002)
(Kubiak 2014)
(Lafaurie 2023)
(Lambe 2016)
(Lambe 2018)
(Mandolfo 2020)
(Mezoff 2016)
(Moore 2014)
(Murray 2014)
(Niño-Serna 2023)
(Öncü 2014)
(Padilla-Orozco 2019)
(Parienti 2014)
(Peterson 2009)
(Piersigilli 2014)
(Piersigilli 2022)
(Pietka 2019)
(Poole 2004)
(Puoti 2023)
(Raphael 2016)
(Santarpia 2002)
(Saxena 2005)
(Silva 2012)
(Silva 2013)
(Simon 2008)
(Smego 1985)
(Souza Dias 2008)
(Tsai 2015)
(Vanegas Calderon 2021)
(Winnett 2008)
Domains:
D1: Bias due to confounding.
D2: Bias due to selection of participants.
D3: Bias in classification of interventions.
D4: Bias due to deviations from intended interventions.
D5: Bias due to missing data.
D6: Bias in measurement of outcomes.
D7: Bias in selection of the reported result.
Judgement
Critical
Serious
Moderate
Low
