# Supplementary material for: Antimicrobial Lock Therapy in Clinical Practice: A Scoping Review
Source: Microorganisms. 2025 Feb 13;13(2):406. doi: 10.3390/microorganisms13020406 (PMC11857916; doi:10.3390/microorganisms13020406)
Supplement: Supplementary file 1 [file microorganisms-13-00406-s001.zip › microorganisms-3411513 Supplementary Matherial Bibliography .pdf]

Supplementary Matherial Bibliography

1. Morales-Molina, J.A.; Mateu-de Antonio, J.; Grau, S.; Segura, M.; Acosta, P. [Stability: a factor to consider in antibiotic-lock solutions]. *Enferm Infecc Microbiol Clin* **2010**, *28*, 104-109, doi:10.1016/j.eimc.2008.09.016.
2. Jaffer, Y.; Selby, N.M.; Taal, M.W.; Fluck, R.J.; McIntyre, C.W. A meta-analysis of hemodialysis catheter locking solutions in the prevention of catheter-related infection. *Am J Kidney Dis* **2008**, *51*, 233-241, doi:10.1053/j.ajkd.2007.10.038.
3. Haq, A.; Patel, D.; Gutlapalli, S.D.; Hernandez, G.N.; Seffah, K.D.; Zaman, M.A.; Awais, N.; Satnarine, T.; Ahmed, A.; Khan, S. A Systematic Review of the Impact of Antibiotic and Antimicrobial Catheter Locks on Catheter-Related Infections in Adult Patients Receiving Hemodialysis. *Cureus* **2023**, *15*, e45000, doi:10.7759/cureus.45000.
4. Mermel, L.A.; Alang, N. Adverse effects associated with ethanol catheter lock solutions: a systematic review. *J Antimicrob Chemother* **2014**, *69*, 2611-2619, doi:10.1093/jac/dku182.
5. Schoot, R.A.; van Dalen, E.C.; van Ommen, C.H.; van de Wetering, M.D. Antibiotic and other lock treatments for tunnelled central venous catheter-related infections in children with cancer. *Cochrane Database Syst Rev* **2013**, *2013*, CD008975, doi:10.1002/14651858.CD008975.pub2.
6. Taylor, J.E.; Tan, K.; Lai, N.M.; McDonald, S.J. Antibiotic lock for the prevention of catheter-related infection in neonates. *Cochrane Database Syst Rev* **2015**, *2015*, CD010336, doi:10.1002/14651858.CD010336.pub2.
7. Blair, N.; Patil, P.; Nguyen, D.; Paudyal-Nepal, B.; Iorember, F. Antibiotic lock solutions as adjunct therapy for catheter-related blood stream infections in pediatric hemodialysis patients. *Front Pediatr* **2024**, *12*, 1379895, doi:10.3389/fped.2024.1379895.
8. Alsultan, M.; Kliea, M.; Hassan, Q. Antibiotic Lock Therapy (ALT) for the prevention of Catheter Related Blood Stream Infection (CRBSI) in neurological patients treated with Therapeutic Plasma Exchange (TPE). *IDCases* **2024**, *37*, e02054, doi:10.1016/j.idcr.2024.e02054.
9. Snaterse, M.; Ruger, W.; Scholte Op Reimer, W.J.; Lucas, C. Antibiotic-based catheter lock solutions for prevention of catheter-related bloodstream infection: a systematic review of randomised controlled trials. *J Hosp Infect* **2010**, *75*, 1-11, doi:10.1016/j.jhin.2009.12.017.
10. Liu, J.; Wang, C.; Zhao, H.; Zhang, J.; Ma, J.; Hou, Y.; Zou, H. Anticoagulant therapies versus heparin for the prevention of hemodialysis catheter-related complications: systematic review and meta-analysis of prospective randomized controlled trials. *Int J Clin Exp Med* **2015**, *8*, 11985-11995.
11. O'Horo, J.C.; Silva, G.L.; Safdar, N. Anti-infective locks for treatment of central line-associated bloodstream infection: a systematic review and meta-analysis. *Am J Nephrol* **2011**, *34*, 415-422, doi:10.1159/000331262.
12. Zacharioudakis, I.M.; Zervou, F.N.; Arvanitis, M.; Ziakas, P.D.; Mermel, L.A.; Mylonakis, E. Antimicrobial lock solutions as a method to prevent central line-associated bloodstream infections: a meta-analysis of randomized controlled trials. *Clin Infect Dis* **2014**, *59*, 1741-1749, doi:10.1093/cid/ciu671.
13. Arechabala, M.C.; Catoni, M.I.; Claro, J.C.; Rojas, N.P.; Rubio, M.E.; Calvo, M.A.; Letelier, L.M. Antimicrobial lock solutions for preventing catheter-related infections in haemodialysis. *Cochrane Database Syst Rev* **2018**, *4*, CD010597, doi:10.1002/14651858.CD010597.pub2.
14. Zhang, J.; Li, R.K.; Chen, K.H.; Ge, L.; Tian, J.H. Antimicrobial lock solutions for the prevention of catheter-related infection in patients undergoing haemodialysis: study protocol for network meta-analysis of randomised controlled trials. *BMJ Open* **2016**, *6*, e010264, doi:10.1136/bmjopen-2015-010264.
15. Yahav, D.; Rozen-Zvi, B.; Gafter-Gvili, A.; Leibovici, L.; Gafter, U.; Paul, M. Antimicrobial lock solutions for the prevention of infections associated with intravascular catheters in patients

undergoing hemodialysis: systematic review and meta-analysis of randomized, controlled trials. *Clin Infect Dis* **2008**, *47*, 83-93, doi:10.1086/588667.
16. Lai, N.M.; Chaiyakunapruk, N.; Lai, N.A.; O'Riordan, E.; Pau, W.S.; Saint, S. Catheter impregnation, coating or bonding for reducing central venous catheter-related infections in adults. *Cochrane Database Syst Rev* **2016**, *3*, CD007878, doi:10.1002/14651858.CD007878.pub3.
17. Schiffer, C.A.; Mangu, P.B.; Wade, J.C.; Camp-Sorrell, D.; Cope, D.G.; El-Rayes, B.F.; Gorman, M.; Ligibel, J.; Mansfield, P.; Levine, M. Central venous catheter care for the patient with cancer: American Society of Clinical Oncology clinical practice guideline. *J Clin Oncol* **2013**, *31*, 1357-1370, doi:10.1200/JCO.2012.45.5733.
18. Christison-Lagay, E.R.; Brown, E.G.; Bruny, J.; Funaro, M.; Glick, R.D.; Dasgupta, R.; Grant, C.N.; Engwall-Gill, A.J.; Lautz, T.B.; Rothstein, D.; et al. Central Venous Catheter Consideration in Pediatric Oncology: A Systematic Review and Meta-analysis From the American Pediatric Surgical Association Cancer Committee. *J Pediatr Surg* **2024**, *59*, 1427-1443, doi:10.1016/j.jpedsurg.2024.03.047.
19. Zhao, Y.; Li, Z.; Zhang, L.; Yang, J.; Yang, Y.; Tang, Y.; Fu, P. Citrate versus heparin lock for hemodialysis catheters: a systematic review and meta-analysis of randomized controlled trials. *Am J Kidney Dis* **2014**, *63*, 479-490, doi:10.1053/j.ajkd.2013.08.016.
20. Sheng, K.X.; Zhang, P.; Li, J.W.; Cheng, J.; He, Y.C.; Bohlke, M.; Chen, J.H. Comparative efficacy and safety of lock solutions for the prevention of catheter-related complications including infectious and bleeding events in adult haemodialysis patients: a systematic review and network meta-analysis. *Clin Microbiol Infect* **2020**, *26*, 545-552, doi:10.1016/j.cmi.2019.12.003.
21. Dang, F.P.; Li, H.J.; Wang, R.J.; Wu, Q.; Chen, H.; Ren, J.J.; Tian, J.H. Comparative efficacy of various antimicrobial lock solutions for preventing catheter-related bloodstream infections: A network meta-analysis of 9099 patients from 52 randomized controlled trials. *Int J Infect Dis* **2019**, *87*, 154-165, doi:10.1016/j.ijid.2019.08.017.
22. Gompelman, M.; Paus, C.; Bond, A.; Akkermans, R.P.; Bleeker-Rovers, C.P.; Lal, S.; Wanten, G.J.A. Comparing success rates in central venous catheter salvage for catheter-related bloodstream infections in adult patients on home parenteral nutrition: a systematic review and meta-analysis. *Am J Clin Nutr* **2021**, *114*, 1173-1188, doi:10.1093/ajcn/nqab164.
23. Calabrese, V.; Farina, A.; Maressa, V.; Cernaro, V.; Gembillo, G.; Messina, R.M.; Longhitano, E.; Ferio, C.; Venanzi Rullo, E.; Santoro, D. Differences in Efficacy between Antibacterial Lock Therapy and the Standard of Care for CVC-Related Infections: A Systematic Review and Meta-Analysis. *Clin Pract* **2024**, *14*, 1538-1549, doi:10.3390/clinpract14040124.
24. Jimenez, M.; Madrid, T. Does antibiotic lock therapy prevent catheter-associated bacteremia in hemodialysis? *Medwave* **2015**, *15*, e6069, doi:10.5867/medwave.2015.01.6069.
25. Zhang, J.; Wang, B.; Li, R.; Ge, L.; Chen, K.H.; Tian, J. Does antimicrobial lock solution reduce catheter-related infections in hemodialysis patients with central venous catheters? A Bayesian network meta-analysis. *Int Urol Nephrol* **2017**, *49*, 701-716, doi:10.1007/s11255-016-1490-x.
26. Yakasai, A.M.; Muhammad, H.; Iliyasu, G.; Nalado, A.M.; Dalhat, M.M.; Habib, Z.G.; Daiyabu, F.; Edwin, C.P.; Maiyaki, M.B.; Ibrahim, D.A. Efficacy of antimicrobial lock solutions in preventing catheter-related blood stream infection in haemodialysis patients: a systematic review and meta-analysis of prospective randomised controlled trials. *Southern African Journal of Infectious Diseases* **2016**, *31*, 95-102, doi:10.1080/23120053.2016.1156811.
27. Sierra, C.; Bahjri, K. 2021 ACCP VIRTUAL Annual Meeting October 19 - 20, 2021. *Jaccp: Journal of the American College of Clinical Pharmacy* **2021**, *4*, 1631-1755, doi:10.1002/jac5.1561.
28. Sierra, C.M.; Rodriquez, C.; Bahjri, K. Ethanol Lock for Prevention of CVC-Related Bloodstream Infection in Pediatric Patients: A Systematic Review and Meta-Analysis. *J Pediatr Pharmacol Ther* **2023**, *28*, 386-396, doi:10.5863/1551-6776-28.5.386.
29. Oliveira, C.; Nasr, A.; Brindle, M.; Wales, P.W. Abstracts. *Pediatric Transplantation* **2011**, *15*, 41-139, doi:10.1111/j.1399-3046.2011.01525.x.

30. Zhang, J.; Wang, B.; Wang, J.; Yang, Q. Ethanol locks for the prevention of catheter-related infection in patients with central venous catheter: A systematic review and meta-analysis of randomized controlled trials. *PLoS One* **2019**, *14*, e0222408, doi:10.1371/journal.pone.0222408.
31. Oliveira, C.; Nasr, A.; Brindle, M.; Wales, P.W. Ethanol locks to prevent catheter-related bloodstream infections in parenteral nutrition: a meta-analysis. *Pediatrics* **2012**, *129*, 318-329, doi:10.1542/peds.2011-1602.
32. Mermel, L.A.; Farr, B.M.; Sherertz, R.J.; Raad, II; O'Grady, N.; Harris, J.S.; Craven, D.E.; Infectious Diseases Society of America, A.C.o.C.C.M.S.f.H.E.o.A. Guidelines for the management of intravascular catheter-related infections. *J Intraven Nurs* **2001**, *24*, 180-205.
33. James, M.T.; Conley, J.; Tonelli, M.; Manns, B.J.; MacRae, J.; Hemmelgarn, B.R.; Alberta Kidney Disease, N. Meta-analysis: antibiotics for prophylaxis against hemodialysis catheter-related infections. *Ann Intern Med* **2008**, *148*, 596-605, doi:10.7326/0003-4819-148-8-200804150-00004.
34. Durning, S. Multidisciplinary team approach to develop aiorithms to guide clinical practice for key issues in pediatric vascular access. *JAVA - Journal of the Association for Vascular Access* **2011**, *16*, 214-215.
35. Takashima, M.; Ezure, Y.; Furuya-Kanamori, L.; Wolf, J.; Dufficy, M.; Gibson, V.; Clark, J.; Ullman, A. Pediatric Central Venous Access Device Lock Solutions: A Network Meta-analysis. *Pediatrics* **2024**, *153*, doi:10.1542/peds.2023-063264.
36. Liu, H.; Liu, H.; Deng, J.; Chen, L.; Yuan, L.; Wu, Y. Preventing catheter-related bacteremia with taurolidine-citrate catheter locks: a systematic review and meta-analysis. *Blood Purif* **2014**, *37*, 179-187, doi:10.1159/000360271.
37. Labriola, L.; Crott, R.; Jadoul, M. Preventing haemodialysis catheter-related bacteraemia with an antimicrobial lock solution: a meta-analysis of prospective randomized trials. *Nephrol Dial Transplant* **2008**, *23*, 1666-1672, doi:10.1093/ndt/gfm847.
38. McGrath, E.J.; Salloum, R.; Chen, X.; Jiang, Y.; Boldt-MacDonald, K.; Becker, C.; Chu, R.; Ang, J.Y. Short-dwell ethanol lock therapy in children is associated with increased clearance of central line-associated bloodstream infections. *Clin Pediatr (Phila)* **2011**, *50*, 943-951, doi:10.1177/0009922811409568.
39. Huang, E.Y.; Chen, C.; Abdullah, F.; Aspelund, G.; Barnhart, D.C.; Calkins, C.M.; Cowles, R.A.; Downard, C.D.; Goldin, A.B.; Lee, S.L.; et al. Strategies for the prevention of central venous catheter infections: an American Pediatric Surgical Association Outcomes and Clinical Trials Committee systematic review. *J Pediatr Surg* **2011**, *46*, 2000-2011, doi:10.1016/j.jpedsurg.2011.06.017.
40. Abdul Salim, S.; Masoud, A.T.; Thongprayoon, C.; Cheungpasitporn, W.; Soliman, K.M.; Garla, V.; Sofy, A.A.; Ahmed, A.S.; Abdelsattar, A.T.; Zsom, L.; et al. Systematic Review and Meta-Analysis of Antibiotic and Antimicrobial Lock Solutions for Prevention of Hemodialysis Catheter-Related Infections. *ASAIO J* **2021**, *67*, 1079-1086, doi:10.1097/MAT.0000000000001382.
41. Rahhal, R.; Abu-El-Haija, M.A.; Fei, L.; Ebach, D.; Orkin, S.; Kiscaden, E.; Cole, C.R. Systematic Review and Meta-Analysis of the Utilization of Ethanol Locks in Pediatric Patients With Intestinal Failure. *JPEN J Parenter Enteral Nutr* **2018**, *42*, 690-701, doi:10.1177/0148607117722753.
42. Gibson, B.; McNiven, C.; Sebastianski, M.; Vandermeer, B.; Persad, R.; Robinson, J.L. Systematic Review of Antimicrobial Lock Solutions for Prevention of Bacteremia in Pediatric Patients With Intestinal Failure. *J Pediatr Gastroenterol Nutr* **2023**, *76*, 410-417, doi:10.1097/MPG.0000000000003658.
43. Norris, L.B.; Kablaoui, F.; Brilhart, M.K.; Bookstaver, P.B. Systematic review of antimicrobial lock therapy for prevention of central-line-associated bloodstream infections in adult and pediatric cancer patients. *Int J Antimicrob Agents* **2017**, *50*, 308-317, doi:10.1016/j.ijantimicag.2017.06.013.

44. Liu, Y.; Zhang, A.Q.; Cao, L.; Xia, H.T.; Ma, J.J. Taurolidine lock solutions for the prevention of catheter-related bloodstream infections: a systematic review and meta-analysis of randomized controlled trials. *PLoS One* **2013**, *8*, e79417, doi:10.1371/journal.pone.0079417.
45. van den Bosch, C.H.; Jeremiasse, B.; van der Bruggen, J.T.; Frakking, F.N.J.; Loeffen, Y.G.T.; van de Ven, C.P.; van der Steeg, A.F.W.; Fiocco, M.F.; van de Wetering, M.D.; Wijnen, M. The efficacy of taurolidine containing lock solutions for the prevention of central-venous-catheter-related bloodstream infections: a systematic review and meta-analysis. *J Hosp Infect* **2022**, *123*, 143-155, doi:10.1016/j.jhin.2021.10.022.
46. Goh, T.L.; Wei, J.; Semple, D.; Collins, J. The incidence and costs of bacteremia due to lack of gentamicin lock solutions for dialysis catheters. *Nephrology (Carlton)* **2017**, *22*, 485-489, doi:10.1111/nep.12960.
47. Safdar, N.; Maki, D.G. Use of vancomycin-containing lock or flush solutions for prevention of bloodstream infection associated with central venous access devices: a meta-analysis of prospective, randomized trials. *Clin Infect Dis* **2006**, *43*, 474-484, doi:10.1086/505976.
48. Dang, F.; Li, H.; Tian, J.; Wang, R.; Ren, J. What is the best catheter lock solution in preventing catheter-related blood infections? A protocol for a Bayesian network meta-analysis of randomised controlled trials. *BMJ Open* **2019**, *9*, e030019, doi:10.1136/bmjopen-2019-030019.
49. Corbella, M.; Brandolini, M.; Cambieri, P.; Decembrino, N.; Pagani, M.; Bottazzi, A.; Muzzi, A.; Zecca, M.; Mariani, B.; Marone, P. A catheter-related bloodstream infection caused by Chryseobacterium indologenes successfully treated with antibiotic-lock rescue therapy. *New Microbiol* **2017**, *40*, 223-225.
50. Sarı, N.; Okur, N.; Çakmakcı, S.; Aksu, T.; İlhan, İ.E. Antibiotic Lock Therapy with Linezolid for the Treatment of Persistent Catheter-Related Infection in Children with Cancer. *Journal of Pediatric Infectious Diseases* **2020**, *15*, 238-241, doi:10.1055/s-0040-1712922.
51. Grau, S.; Gil, M.J.; Mateu-de Antonio, J.; Pera, M.; Marin-Casino, M. Antibiotic-lock technique using daptomycin for subcutaneous injection ports in a patient on home parenteral nutrition. *J Infect* **2009**, *59*, 298-299, doi:10.1016/j.jinf.2009.08.003.
52. Koparkar, V.; Soman, R.; Shetty, A.; Purandare, B.; Sunavala, A.; Doshi, A. P011 Antifungal lock therapy. *Medical Mycology* **2022**, *60*, doi:10.1093/mmy/myac072.P011.
53. Bregenzer, T.; Widmer, A.F. Bloodstream infection from a Port-A-Cath: successful treatment with the antibiotic lock technique. *Infect Control Hosp Epidemiol* **1996**, *17*, 772, doi:10.1086/647231.
54. Angel-Moreno, A.; Boronat, M.; Bolanos, M.; Carrillo, A.; Gonzalez, S.; Perez Arellano, J.L. Candida glabrata fungemia cured by antibiotic-lock therapy: case report and short review. *J Infect* **2005**, *51*, e85-87, doi:10.1016/j.jinf.2004.08.034.
55. Castagnola, E.; Moroni, C.; Gandullia, P.; Oddone, M.; Peri, C.; Casciaro, R.; De Alessandri, A. Catheter lock and systemic infusion of linezolid for treatment of persistent Broviac catheter-related staphylococcal bacteremia. *Antimicrob Agents Chemother* **2006**, *50*, 1120-1121, doi:10.1128/AAC.50.3.1120-1121.2006.
56. Chuang, C.Y.; Yang, Y.L.; Hsueh, P.R.; Lee, P.I. Catheter-related bacteremia caused by Staphylococcus pseudintermedius refractory to antibiotic-lock therapy in a hemophilic child with dog exposure. *J Clin Microbiol* **2010**, *48*, 1497-1498, doi:10.1128/JCM.02033-09.
57. Rossi, L.; Libutti, P.; Casucci, F.; Lisi, P.; Corciulo, S.; Lomonte, C. Sp582catheter-Related Right Atrial Thrombosis Treated with Systemic Anticoagulation and Loco-Regional Thrombolytic Therapy without Catheter Removal. *Nephrology Dialysis Transplantation* **2019**, *34*, doi:10.1093/ndt/gfz103.SP582.
58. Wong, T.; Clifford, V.; McCallum, Z.; Shalley, H.; Peterkin, M.; Paxton, G.; Bines, J.E. Central venous catheter thrombosis associated with 70% ethanol locks in pediatric intestinal failure patients on home parenteral nutrition: a case series. *JPEN J Parenter Enteral Nutr* **2012**, *36*, 358-360, doi:10.1177/0148607111414713.

59. Sadyrbaeva-Dolgova, S.; García-Valdes, M.; Nucete-Gallego, B.; Villegas-Gongora, D.; Hernandez-Magdalena, J. Abstracts of 48th ESCP symposium on clinical pharmacy 23-25 October 2019, Ljubljana (Slovenia) : The digital revolution supporting clinical pharmacy through e-health, digital support systems, big data, and more. *Int J Clin Pharm* **2019**, *42*, 217-292, doi:10.1007/s11096-019-00945-w.
60. Pieroni, K.P.; Nespor, C.; Poole, R.L.; Kerner, J.A., Jr.; Berquist, W.E. Echinocandin and ethanol lock therapy treatment of fungal catheter infections. *Pediatr Infect Dis J* **2013**, *32*, 289-291, doi:10.1097/INF.0b013e3182784867.
61. Brayer, S.; Linn, A.; Holt, S.; Ellery, K.; Mitchell, S.; Williams, J. Enterococcus hirae Bacteremia in an Infant: Case Report and Review of the Literature. *J Pediatric Infect Dis Soc* **2019**, *8*, 571-573, doi:10.1093/jpids/piz028.
62. Ackoundou-N'guessan, C.; Heng, A.E.; Guenu, S.; Charbonne, F.; Traore, O.; Deteix, P.; Souweine, B. Ethanol lock solution as an adjunct treatment for preventing recurrent catheter-related sepsis--first case report in dialysis setting. *Nephrol Dial Transplant* **2006**, *21*, 3339-3340, doi:10.1093/ndt/gfl358.
63. Rajpurkar, M.; Boldt-Macdonald, K.; McLenon, R.; Callaghan, M.U.; Chitlur, M.; Lusher, J.M.; Becker, C. Ethanol lock therapy for the treatment of catheter-related infections in haemophilia patients. *Haemophilia* **2009**, *15*, 1267-1271, doi:10.1111/j.1365-2516.2009.02075.x.
64. Blackwood, R.A.; Klein, K.C.; Micel, L.N.; Willers, M.L.; Mody, R.J.; Teitelbaum, D.H.; Cober, M.P. Ethanol locks therapy for resolution of fungal catheter infections. *Pediatr Infect Dis J* **2011**, *30*, 1105-1107, doi:10.1097/INF.0b013e31822c84a4.
65. Pieroni, K.P.; Nespor, C.; Ng, M.; Garcia, M.; Hurwitz, M.; Berquist, W.E.; Kerner, J.A., Jr. Evaluation of ethanol lock therapy in pediatric patients on long-term parenteral nutrition. *Nutr Clin Pract* **2013**, *28*, 226-231, doi:10.1177/0884533612468009.
66. Guimard, T.; Revest, M.; Arvieux, C.; Michelet, C.; Tattevin, P.; Donnio, P.Y.; Tribut, O. Failure of antibiotic-lock technique using daptomycin for subcutaneous injection ports in a patient on home parenteral nutrition. *J Infect* **2010**, *60*, 505-507, doi:10.1016/j.jinf.2010.03.022.
67. Puapatanakul, P.; Lumlertgul, N.; Thongbor, N.; Mahamongkholsawat, J.; Kanjanabuch, T. Intracatheter Antifungal Lock Leading to Detrimental Complications. *Med Mycol Case Rep* **2018**, *22*, 58-60, doi:10.1016/j.mmcr.2018.09.001.
68. Barrie, A.; Efford, J.; Spires, J.; Mathias, M.; Sibson, K.; Liesner, R.; Khair, K. Poster Presentations. *Haemophilia* **2017**, *23*, 29-140, doi:10.1111/hae.13150.
69. Colvine, J.; Thomson, P.; Duerksen, D.R. Management of Recurrent Catheter-Related Bloodstream Infections in an Adult Patient Receiving Home Parenteral Nutrition: Dramatic Effect of Ethanol Lock Therapy. *JPEN J Parenter Enteral Nutr* **2017**, *41*, 1072-1074, doi:10.1177/0148607115602890.
70. Gattuso, G.; Tomasoni, D.; Ceruti, R.; Scalzini, A. Multiresistant Stenotrophomonas maltophilia tunneled CVC-related sepsis, treated with systemic and lock therapy. *J Chemother* **2004**, *16*, 494-496, doi:10.1179/joc.2004.16.5.494.
71. Elwood, R.L.; Spencer, S.E. Successful clearance of catheter-related bloodstream infection by antibiotic lock therapy using ampicillin. *Ann Pharmacother* **2006**, *40*, 347-350, doi:10.1345/aph.1G446.
72. Vandenhende, M.A.; Buret, J.; Camou, F.; Morlat, P.; Bonnet, F. Successful daptomycin lock therapy for implantable intra-arterial catheter infection in a patient with liver metastases of colon cancer. *Diagn Microbiol Infect Dis* **2014**, *78*, 497-498, doi:10.1016/j.diagmicrobio.2014.01.006.
73. Volakli, E.; Violaki, A.; Svirkos, M.; Chochliourou, E.; Karakeke, E.; Anastasiou, A.; Kalaitzi, A.; Georgiadou, P.; Kotzapanagiotou, E.; Sdougka, M. EAP 2019 Congress and Master Course. *European Journal of Pediatrics* **2019**, *178*, 1613-1800, doi:10.1007/s00431-019-03466-w.
74. Bartalesi, F.; Veloci, S.; Baragli, F.; Mantengoli, E.; Guidi, S.; Bartolesi, A.M.; Mannino, R.; Pecile, P.; Bartoloni, A. Successful tigecycline lock therapy in a Lactobacillus rhamnosus

catheter-related bloodstream infection. *Infection* **2012**, *40*, 331-334, doi:10.1007/s15010-011-0196-3.
75. Ozdemir, H.; Karbuz, A.; Ciftci, E.; Dincaslan, H.U.; Ince, E.; Aysev, D.; Yavuz, G.; Dogru, U. Successful treatment of central venous catheter infection due to Candida lipolytica by caspofungin-lock therapy. *Mycoses* **2011**, *54*, e647-649, doi:10.1111/j.1439-0507.2010.01964.x.
76. Jurewitsch, B.; Lee, T.; Park, J.; Jeejeebhoy, K. Taurolidine 2% as an antimicrobial lock solution for prevention of recurrent catheter-related bloodstream infections. *JPEN J Parenter Enteral Nutr* **1998**, *22*, 242-244, doi:10.1177/0148607198022004242.
77. Li Cavoli, G.; Schillaci, O.; Zagarrigo, C.; Servillo, F.; Li Cavoli, T.V.; Palmeri, M.; Rotolo, U. The urokinase lock-therapy for hemodialysis occluded central venous catheters. *Blood Purif* **2015**, *39*, 238, doi:10.1159/000381007.
78. Pisos-Alamo, E.; Hernandez-Cabrera, M.; Sobral-Caraballo, O.; Perez-Arellano, J.L. The use of co-trimoxazole in catheter lock therapy. A report on a difficult case. *Enferm Infecc Microbiol Clin (Engl Ed)* **2018**, *36*, 321-322, doi:10.1016/j.eimc.2017.08.005.
79. Mohandas, A.; Isava-Quintero, A.; Duro, D. Thirty percent ethanol locks ineffective in preventing central line-associated bloodstream infection in pediatric intestinal rehabilitation patients. *JPEN J Parenter Enteral Nutr* **2021**, *45*, 1376-1379, doi:10.1002/jpen.2129.
80. Foresti, S.; Di Bella, S.; Rovelli, A.; Sala, A.; Verna, M.; Bisi, L.; Nisii, C.; Gori, A. Tigecycline Lock Therapy for Catheter-Related Bloodstream Infection Caused by KPC-Producing Klebsiella pneumoniae in Two Pediatric Hematological Patients. *Antimicrob Agents Chemother* **2015**, *59*, 7919-7920, doi:10.1128/AAC.01855-15.
81. Buckler, B.S.; Sams, R.N.; Goei, V.L.; Krishnan, K.R.; Bemis, M.J.; Parker, D.P.; Murray, D.L. Treatment of central venous catheter fungal infection using liposomal amphotericin-B lock therapy. *Pediatr Infect Dis J* **2008**, *27*, 762-764, doi:10.1097/INF.0b013e318170b68b.
82. Xia, C.; Fan, J.; Xu, C.; Hu, S.; Ma, H.; He, L.; Ye, L. Urokinase-based lock solutions for catheter salvage: A case of an upcoming kidney transplant recipient. *Open Med (Wars)* **2023**, *18*, 20230699, doi:10.1515/med-2023-0699.
83. Piersigilli, F.; Auriti, C.; Bersani, I.; Campi, F.; Savarese, I.; Goffredo, B.M.; Dotta, A. Use of Antimicrobial Lock Therapy for the Treatment of Central Line-Related Blood-Stream Infections: A Case Series. *American Journal of Perinatology* **2018/05**, *35*, doi:10.1055/s-0038-1647089.
84. Halleux, D.; Labriola, L.; Jadoul, M. Vancomycin catheter lock as a cause of gross overestimation of vancomycin pre-dialysis trough levels. *Nephrol Dial Transplant* **2011**, *26*, 2061-2062; author reply 2062-2063, doi:10.1093/ndt/gfr062.
85. Brescia, F.; Pittiruti, M.; Scoppettuolo, G.; Zanier, C.; Nadalini, E.; Bottos, P.; Moreal, C.; Da Ros, V.; Fabiani, F. Taurolidine lock in the treatment of colonization and infection of totally implanted venous access devices in cancer patients. *J Vasc Access* **2023**, *24*, 87-91, doi:10.1177/11297298211026453.
86. Caccia, J. Antibiotic lock technique combined with systemic antimicrobial therapy for management of port-related bloodstream infections in onco-haematological patients without catheter removal. Université de Lausanne, Faculté de biologie et médecine, 2011.
87. Chaftari, A.M.; Hachem, R.; Szvalb, A.; Taremi, M.; Granwehr, B.; Viola, G.M.; Amin, S.; Assaf, A.; Numan, Y.; Shah, P.; et al. A Novel Nonantibiotic Nitroglycerin-Based Catheter Lock Solution for Prevention of Intraluminal Central Venous Catheter Infections in Cancer Patients. *Antimicrob Agents Chemother* **2017**, *61*, doi:10.1128/AAC.00091-17.
88. Haag, G.M.; Berger, A.K.; Jager, D. Treatment of long-term catheter-related bloodstream infections with a taurolidine block: a single cancer center experience. *J Vasc Access* **2011**, *12*, 244-247, doi:10.5301/JVA.2011.6265.
89. Hung, F.C.; Liao, C.H. Improvement program for bloodstream infection in an oncology ward. *International Journal of Antimicrobial Agents* **2017**, *50*, S245.

90. Izzo, I.; Lania, D.; Castro, A.; Lanzini, F.; Bella, D.; Pagani, A.; Colombini, P. [Seven cases of port-a-cath contamination caused by Pantoea agglomerans in the Oncological Service of Iseo Hospital, Brescia (Italy)]. *Infez Med* **2014**, *22*, 152-155.
91. Koldehoff, M.; Zakrzewski, J.L. Taurolidine is effective in the treatment of central venous catheter-related bloodstream infections in cancer patients. *Int J Antimicrob Agents* **2004**, *24*, 491-495, doi:10.1016/j.ijantimicag.2004.06.006.
92. McGrath, E.; Du, W.; Rajpurkar, M. Preemptive Ethanol Lock Therapy in Pediatric Hematology/Oncology Patients With Catheter-Associated Bloodstream Infection: Impact on Length of Stay, Cost, and Catheter Salvage. *Clin Pediatr (Phila)* **2018**, *57*, 285-293, doi:10.1177/0009922817717327.
93. Vassallo, M.; Denis, E.; Manni, S.; Lotte, L.; Fauque, P.; Sindt, A. Treatment of long-term catheter-related bloodstream infections with short-course Daptomycin lock and systemic therapy associated with Taurolidine-lock: A multicenter experience. *J Vasc Access* **2024**, *25*, 1146-1150, doi:10.1177/11297298231152500.
94. Zanwar, S.; Jain, P.; Gokarn, A.; Devadas, S.K.; Punatar, S.; Khurana, S.; Bonda, A.; Pruthy, R.; Bhat, V.; Qureshi, S.; et al. Antibiotic lock therapy for salvage of tunneled central venous catheters with catheter colonization and catheter-related bloodstream infection. *Transpl Infect Dis* **2019**, *21*, e13017, doi:10.1111/tid.13017.
95. Kara, T.T.; Ozdemir, H.; Erat, T.; Yahsi, A.; Aysev, A.D.; Tacyildiz, N.; Unal, E.; Ileri, T.; Ince, E.; Haskologlu, S.; et al. Is antibiotic lock therapy effective for the implantable longterm catheter-related bloodstream infections in children? *Turk J Pediatr* **2019**, *61*, 895-904, doi:10.24953/turkjped.2019.06.011.
96. Kurtipek, F.B.; Yozgat, A.K.; Kanik-Yuksek, S.; Kacar, D.; Bayhan, T.; Gokcebay, D.G.; Parlakay, A.O.; Yarali, N. Antibiotic Lock Therapy for Port Catheter-Related Infections of Children with Acute Leukemia. *Mediterr J Hematol Infect Dis* **2024**, *16*, e2024072, doi:10.4084/MJHID.2024.072.
97. Miliaraki, M.; Katzilakis, N.; Chranioti, I.; Stratigaki, M.; Koutsaki, M.; Psarrou, M.; Athanasopoulos, E.; Stiakaki, E. Central line-associated bloodstream infection in childhood malignancy: Single-center experience. *Pediatr Int* **2017**, *59*, 769-775, doi:10.1111/ped.13289.
98. Plourde, R.; Gothard, D.; Markowski, A.; Cockrell, E. 2011 ASPHO Abstracts. *Pediatric Blood & Cancer* **2011**, *56*, 897-973, doi:10.1002/pbc.23092.
99. Qureshi, S.; Fatima, P.; Mukhtar, A.; Zehra, A.; Qamar, F.N. Clinical profile and outcome of antibiotic lock therapy for bloodstream infections in pediatric hematology/oncology patients in a tertiary care hospital, Karachi, Pakistan. *Int J Pediatr Adolesc Med* **2019**, *6*, 25-28, doi:10.1016/j.ijpam.2019.01.004.
100. Salloum, R.; McGrath, E.; Chen, X.; Becker, C.; Boldt-Macdonald, K.; Ang, J.; Chu, R. American Society of Pediatric Hematology/Oncology 23rd Annual Meeting: Abstracts. *Pediatric Blood & Cancer* **2010**, *54*, 787-864, doi:10.1002/pbc.22516.
101. Glatstein, E.; Bertoni, L.; Garnero, A.; Vanzo, C.; Gomila, A. Antibiotic-Lock Therapy in Pediatric Oncology Patients. *Journal of Pediatric Infectious Diseases* **2015**, *10*, 039-044, doi:10.1055/s-0035-1564067.
102. Walker, L.W.; Nowalk, A.J. 2856. Salvaging High-Value Catheters: Antifungal Lock Therapy for Candidal Central Catheter Infections in a Pediatric Cohort. *Open Forum Infectious Diseases* **2019**, *6*, S75-S76, doi:10.1093/ofid/ofz359.161.
103. Blackwood, R.A.; Issa, M.; Klein, K.; Mody, R.; Willers, M.; Teitelbaum, D. Ethanol Lock Therapy for the Treatment of Intravenous Catheter Infections That Have Failed Standard Treatment. *J Pediatric Infect Dis Soc* **2017**, *6*, 94-97, doi:10.1093/jpids/piv060.
104. Krzywda, E.A.; Andris, D.A.; Edmiston, C.E., Jr.; Quebbeman, E.J. Treatment of Hickman catheter sepsis using antibiotic lock technique. *Infect Control Hosp Epidemiol* **1995**, *16*, 596-598, doi:10.1086/647015.
105. Tena, D.; Carranza, R.; Barbera, J.R.; Valdezate, S.; Garrancho, J.M.; Arranz, M.; Saez-Nieto, J.A. Outbreak of long-term intravascular catheter-related bacteremia due to Achromobacter

xylosoxidans subspecies xylosoxidans in a hemodialysis unit. *Eur J Clin Microbiol Infect Dis* **2005**, *24*, 727-732, doi:10.1007/s10096-005-0028-4.
106. Aslam, S.; Trautner, B.W.; Ramanathan, V.; Darouiche, R.O. Pilot trial of N-acetylcysteine and tigecycline as a catheter-lock solution for treatment of hemodialysis catheter-associated bacteremia. *Infect Control Hosp Epidemiol* **2008**, *29*, 894-897, doi:10.1086/590192.
107. Erb, S.; Widmer, A.F.; Tschudin-Sutter, S.; Neff, U.; Fischer, M.; Dickenmann, M.; Grosse, P. Impact of different catheter lock strategies on bacterial colonization of permanent central venous hemodialysis catheters. *Infect Control Hosp Epidemiol* **2013**, *34*, 1314-1317, doi:10.1086/673987.
108. Feely, T.; Copley, A.; Bleyer, A.J. Catheter lock solutions to prevent bloodstream infections in high-risk hemodialysis patients. *Am J Nephrol* **2007**, *27*, 24-29, doi:10.1159/000098541.
109. Garcia, M.C.; Lluch, M.G.; Vega, B.P.; Benito, M.H.; Serrano, J.S.; Gamez, J.V.; Quiñones, J.G. CP-058 Analysis of changes in management of catheter related infections in haemodialysis after implementation of a protocol. *European Journal of Hospital Pharmacy* **2017-03-01**, *24*, doi:10.1136/ejhpharm-2017-000640.57.
110. Mai, H.; Zhao, Y.; Salerno, S.; Li, Y.; Feng, Y.; Ma, L.; Fu, P. Citrate versus heparin lock for prevention of hemodialysis catheter-related complications: updated systematic review and meta-analysis of randomized controlled trials. *Int Urol Nephrol* **2019**, *51*, 1019-1033, doi:10.1007/s11255-019-02150-0.
111. Nguyen, T.; Camins, B.C.; Butler, D.A. Taurolidine and Heparin as Catheter Lock Solution for Central Venous Catheters in Hemodialysis. *Am J Ther* **2024**, *31*, e398-e409, doi:10.1097/MJT.0000000000001736.
112. Patel, J.; Gang, S.; Rajapurkar, M.; Hegde, U.; Konnur, A.; Patel, H. 71. SHORT-DWELL DAILY ETHANOL-LOCK THERAPY IN MANAGEMENT OF HEMODIALYSIS TUNNELED CUFFED CATHETER RELATED BLOOD STREAM INFECTION. *Indian Journal of Nephrology* **2018**, *28*.
113. Quarello, F.; Forneris, G. Prevention of hemodialysis catheter-related bloodstream infection using an antimicrobial lock. *Blood Purif* **2002**, *20*, 87-92, doi:10.1159/000046990.
114. van Eck van der Sluijs, A.; Eekelschot, K.Z.; Frakking, F.N.; Haas, P.A.; Boer, W.H.; Abrahams, A.C. Salvage of the peritoneal dialysis catheter in Candida peritonitis using amphotericin B catheter lock. *Perit Dial Int* **2021**, *41*, 110-114, doi:10.1177/0896860820923238.
115. van Roeden, S.; van Oevelen, M.; Abrahams, A.C.; Dekker, F.W.; Rotmans, J.I.; Meijvis, S.C.A.; group, D.s. The best solution down the line: an observational study on taurolidine- versus citrate-based lock solutions for central venous catheters in hemodialysis patients. *BMC Nephrol* **2021**, *22*, 308, doi:10.1186/s12882-021-02519-3.
116. Venditto, M.; du Montcel, S.T.; Robert, J.; Trystam, D.; Dighiero, J.; Hue, D.; Bessette, C.; Deray, G.; Mercadal, L. Effect of catheter-lock solutions on catheter-related infection and inflammatory syndrome in hemodialysis patients: heparin versus citrate 46% versus heparin/gentamicin. *Blood Purif* **2010**, *29*, 268-273, doi:10.1159/000274461.
117. Del Risco Zevallos, J.; Molina Andujar, A.; Pineiro, G.; Morata, L.; Casals, J.; Jimenez, M.; Fontsere, N.; Maduell, F.; Soriano, A.; Poch, E. Reduction of hemodialysis catheter-related blood stream infections in intensive care units after systematic use of taurolidine-citrate-heparin locking solution. *J Vasc Access* **2024**, 11297298241282370, doi:10.1177/11297298241282370.
118. Dixon, J.J.; Steele, M.; Makanjuola, A.D. Anti-microbial locks increase the prevalence of Staphylococcus aureus and antibiotic-resistant Enterobacter: observational retrospective cohort study. *Nephrol Dial Transplant* **2012**, *27*, 3575-3581, doi:10.1093/ndt/gfs081.
119. Arnoriaga Rodriguez, M.; Perez de Ciriza Cordeu, M.; Camblor Alvarez, M.; Breton Lesmes, I.; Motilla de la Camara, M.; Velasco Gimeno, C.; Arhip, L.; Garcia Peris, P.; Cuerda Compes, C. Clinical and economic impact of the taurolidine lock on home parenteral nutrition. *Nutr Hosp* **2018**, *35*, 761-766, doi:10.20960/nh.1748.
120. Corrigan, M.L.; Pogatschnik, C.; Konrad, D.; Kirby, D.F. Hospital readmissions for catheter-related bloodstream infection and use of ethanol lock therapy: comparison of patients

receiving parenteral nutrition or intravenous fluids in the home vs a skilled nursing facility. *JPEN J Parenter Enteral Nutr* **2013**, *37*, 81-84, doi:10.1177/0148607112448400.
121. Fernandez-Hidalgo, N.; Almirante, B.; Calleja, R.; Ruiz, I.; Planes, A.M.; Rodriguez, D.; Pigrau, C.; Pahissa, A. Antibiotic-lock therapy for long-term intravascular catheter-related bacteraemia: results of an open, non-comparative study. *J Antimicrob Chemother* **2006**, *57*, 1172-1180, doi:10.1093/jac/dkl103.
122. Fortun, J.; Grill, F.; Martin-Davila, P.; Blazquez, J.; Tato, M.; Sanchez-Corral, J.; Garcia-San Miguel, L.; Moreno, S. Treatment of long-term intravascular catheter-related bacteraemia with antibiotic-lock therapy. *J Antimicrob Chemother* **2006**, *58*, 816-821, doi:10.1093/jac/dkl318.
123. Guedon, C.; Nouvellon, M.; Lalaude, O.; Lerebours, E. Efficacy of antibiotic-lock technique with teicoplanin in staphylococcus epidermidis catheter-related sepsis during long-term parenteral nutrition. *JPEN J Parenter Enteral Nutr* **2002**, *26*, 109-113, doi:10.1177/0148607102026002109.
124. Hill, J.; Garner, R. Efficacy of 4% tetrasodium ethylenediaminetetraacetic acid (T-EDTA) catheter lock solution in home parenteral nutrition patients: A quality improvement evaluation. *J Vasc Access* **2021**, *22*, 533-539, doi:10.1177/1129729820946916.
125. John, B.K.; Khan, M.A.; Speerhas, R.; Rhoda, K.; Hamilton, C.; Dechicco, R.; Lopez, R.; Steiger, E.; Kirby, D.F. Ethanol lock therapy in reducing catheter-related bloodstream infections in adult home parenteral nutrition patients: results of a retrospective study. *JPEN J Parenter Enteral Nutr* **2012**, *36*, 603-610, doi:10.1177/0148607111428452.
126. John, B.K.; Khan, M.A.; Speerhas, R.; Rhoda, K.; Hamilton, C.; Lopez, R.; Steiger, E.; Kirby, D.F. Ethanol lock therapy in reducing catheter related blood stream infections (CRBSI) in home parenteral nutrition patients. *Gastroenterology* **2010**, *138*, S39.
127. Korzilius, J.W.; Gillis, V.; Wouters, Y.; Wanten, G.J.A. Taurolidine-related adverse events in patients on home parenteral nutrition frequently indicate catheter-related problems. *Clin Nutr* **2022**, *41*, 2178-2184, doi:10.1016/j.clnu.2022.07.025.
128. Lawinski, M.; Majewska, K.; Foltyn, I.; Gradowska, A. The efficacy of alcohol-antibiotic lock therapy for treatment of catheter related bloodstream infections in patients receiving home parenteral nutrition. *Pol Przegl Chir* **2015**, *86*, 563-568, doi:10.1515/pjs-2015-0002.
129. McClelland, J.; Gallotto, M.; Mitchell, P.; Carey, A. Poster Abstracts. *Journal of Parenteral and Enteral Nutrition* **2023**, *47*, S124-S125, doi:10.1002/jpen.2491.
130. McGovern, T.; Stanner, H.; Riddle, E. Poster Abstracts. *Journal of Parenteral and Enteral Nutrition* **2022**, *46*, S115-S116, doi:10.1002/jpen.2345.
131. Messing, B.; Peitra-Cohen, S.; Debure, A.; Beliah, M.; Bernier, J.J. Antibiotic-lock technique: a new approach to optimal therapy for catheter-related sepsis in home-parenteral nutrition patients. *JPEN J Parenter Enteral Nutr* **1988**, *12*, 185-189, doi:10.1177/0148607188012002185.
132. Olthof, E.D.; Huisman-De Waal, G.J.; Versleijen, M.W.; Kievit, W.; Wanten, G.J. UEG Week 2013 Poster Presentations. *United European Gastroenterology Journal* **2013**, *1*, A135-A587, doi:10.1177/2050640613502900.
133. Olthof, E.D.; Rentenaar, R.J.; Rijs, A.J.; Wanten, G.J. Absence of microbial adaptation to taurolidine in patients on home parenteral nutrition who develop catheter related bloodstream infections and use taurolidine locks. *Clin Nutr* **2013**, *32*, 538-542, doi:10.1016/j.clnu.2012.11.014.
134. Olthof, E.D.; Versleijen, M.W.; Huisman-de Waal, G.; Feuth, T.; Kievit, W.; Wanten, G.J. Taurolidine lock is superior to heparin lock in the prevention of catheter related bloodstream infections and occlusions. *PLoS One* **2014**, *9*, e111216, doi:10.1371/journal.pone.0111216.
135. Opilla, M. Peripherally Inserted Central Catheter Experience in Long-Term Home Parenteral Nutrition Patients. *Journal of the Association for Vascular Access* **2017**, *22*, 42-45, doi:10.1016/j.java.2016.12.001.
136. Opilla, M.T.; Kirby, D.F.; Edmond, M.B. Use of ethanol lock therapy to reduce the incidence of catheter-related bloodstream infections in home parenteral nutrition patients. *JPEN J Parenter Enteral Nutr* **2007**, *31*, 302-305, doi:10.1177/0148607107031004302.

137. Sakurai, T.; Nakamura, M.; Sasaki, H.; Fukuzawa, T.; Kudo, H.; Ando, R.; Okubo, R.; Hashimoto, M.; Tada, K.; Wada, M. Risk factors for catheter-related bloodstream infections in patients with intestinal failure undergoing home parenteral nutrition: a single-center study. *Pediatr Surg Int* **2023**, *39*, 283, doi:10.1007/s00383-023-05555-2.
138. Santarpia, L.; Pagano, M.C.; Buonomo, A.; Foggia, M.; Alfonsi, L.; Contaldo, F.; Pasanisi, F. Pp181-Sun Lock Therapy with Daptomycin for the Treatment of Methicillin Resistant S. Epidermidis Infections in Patients on Long-Term Home Parenteral Nutrition. *Clinical Nutrition* **2013**, *32*, doi:10.1016/s0261-5614(13)60226-5.
139. Skorepa, P.; Víšek, J.; Bláha, V.; Fortunato, J.; Sobotka, L. MON-PP010: Retrospective Study of Two Antiseptic Regimens for Catheter-Associated Bloodstream Infection (CBSI) Treatment in Home Parenteral Nutrition Patients. *Clinical Nutrition* **2015**, *34*, S130-S131, doi:10.1016/s0261-5614(15)30442-8.
140. Toure, A.; Lauverjat, M.; Peraldi, C.; Boncompain-Gerard, M.; Gelas, P.; Barnoud, D.; Chambrier, C. Taurolidine lock solution in the secondary prevention of central venous catheter-associated bloodstream infection in home parenteral nutrition patients. *Clin Nutr* **2012**, *31*, 567-570, doi:10.1016/j.clnu.2012.01.001.
141. Wales, P.W.; Kosar, C.; Carricato, M.; de Silva, N.; Lang, K.; Avitzur, Y. Ethanol lock therapy to reduce the incidence of catheter-related bloodstream infections in home parenteral nutrition patients with intestinal failure: preliminary experience. *J Pediatr Surg* **2011**, *46*, 951-956, doi:10.1016/j.jpedsurg.2011.02.036.
142. Worley, M.V.; Dollard, E.W.; Aragon, L.; Henderson, K.; Abbo, L.M.; Byers, P. Role of Ethanol Locks in Reducing Bloodstream Infections in Adults on Parenteral Nutrition. *Infect Control Hosp Epidemiol* **2017**, *38*, 1133-1135, doi:10.1017/ice.2017.154.
143. Wouters, Y.; Roosenboom, B.; Causevic, E.; Kievit, W.; Groenewoud, H.; Wanten, G.J.A. Clinical outcomes of home parenteral nutrition patients using taurolidine as catheter lock: A long-term cohort study. *Clin Nutr* **2019**, *38*, 2210-2218, doi:10.1016/j.clnu.2018.09.020.
144. Young, C.; Allen, P.; Sarkozy, T.; Costlow, L.; Bolton, A.; Heredia, H.; Lloyd, J. Poster Abstracts. *Journal of Parenteral and Enteral Nutrition* **2021**, *45*, S50-S51, doi:10.1002/jpen.2095.
145. Zembles, T.N.; Flannery, L.S.; Huppler, A.R. Development and implementation of an antimicrobial lock therapy guideline in a pediatric hospital. *Am J Health Syst Pharm* **2018**, *75*, 299-303, doi:10.2146/ajhp161056.
146. Bookstaver, P.B.; Gerrald, K.R.; Moran, R.R. Clinical outcomes of antimicrobial lock solutions used in a treatment modality: a retrospective case series analysis. *Clin Pharmacol* **2010**, *2*, 123-130, doi:10.2147/CPAA.S11262.
147. Ornowska, M.; Wong, H.; Ouyang, Y.; Mitra, A.; White, A.; Willems, S.; Wittmann, J.; Reynolds, S. Control of Line Complications with KiteLock (CLiCK) in the critical care unit: study protocol for a multi-center, cluster-randomized, double-blinded, crossover trial investigating the effect of a novel locking fluid on central line complications in the critical care population. *Trials* **2022**, *23*, 719, doi:10.1186/s13063-022-06671-5.
148. Cober, M.P.; Kovacevich, D.S.; Teitelbaum, D.H. Ethanol-lock therapy for the prevention of central venous access device infections in pediatric patients with intestinal failure. *JPEN J Parenter Enteral Nutr* **2011**, *35*, 67-73, doi:10.1177/0148607110362758.
149. Coughlin, M.C.; Ridelman, E.; Lelli, J.L.; Shanti, C.M. Initial blood cultures in pediatric patients with central line infections and short bowel syndrome can direct a treatment plan that can reduce hospital length of stay. *Surgery* **2023**, *173*, 781-787, doi:10.1016/j.surg.2022.07.048.
150. Fligor, S.; Hirsch, T.; Tsikis, S.; Joiner, M.; Mitchell, P.; McClelland, J.; Carey, A.; Gura, K.; Puder, M. Nutrition and Metabolism Research Oral Paper Session Abstracts. *Journal of Parenteral and Enteral Nutrition* **2023**, *47*, S55-S56, doi:10.1002/jpen.2487.
151. Jones, B.A.; Hull, M.A.; Richardson, D.S.; Zurakowski, D.; Gura, K.; Fitzgibbons, S.C.; Duro, D.; Lo, C.W.; Duggan, C.; Jaksic, T. Efficacy of ethanol locks in reducing central venous catheter infections in pediatric patients with intestinal failure. *J Pediatr Surg* **2010**, *45*, 1287-1293, doi:10.1016/j.jpedsurg.2010.02.099.

152. Kleidon, T.; Graham, N.; Clark, J. Abstracts from the 3rd World Congress on Vascular Access, WoCoVA 2014, 18-20 June 2014, Berlin, Germany. *J Vasc Access* **2014**, *15*, 193-239, doi:10.5301/jva.5000275.
153. LaRusso, K.; Dumas, M.P.; Schaack, G.; Sant'Anna, A. Prolonged Use of Ethanol Lock Prophylaxis With Polyurethane Catheters in Children With Intestinal Failure: A Single-Center Experience. *JPEN J Parenter Enteral Nutr* **2021**, *45*, 1425-1431, doi:10.1002/jpen.2056.
154. Ralls, M.W.; Blackwood, R.A.; Arnold, M.A.; Partipilo, M.L.; Dimond, J.; Teitelbaum, D.H. Drug shortage-associated increase in catheter-related blood stream infection in children. *Pediatrics* **2012**, *130*, e1369-1373, doi:10.1542/peds.2011-3894.
155. Abu-El-Haija, M.; Schultz, J.; Rahhal, R.M. Effects of 70% ethanol locks on rates of central line infection, thrombosis, breakage, and replacement in pediatric intestinal failure. *J Pediatr Gastroenterol Nutr* **2014**, *58*, 703-708, doi:10.1097/MPG.0000000000000354.
156. Bharania, B.; Crook, J.; Verma, A. P24 Compliance audit of ethanol linelocks for prophylaxis and treatment of central line infections. *Archives of Disease in Childhood* **2023**, *108*, 13.12-14, doi:10.1136/archdischild-2023-NPPG.23.
157. Carey-Goo, P.V.; Melish, M.; Tadaki, K.; Bankhead, S. Ethanol Lock Therapy for Central Venous Lines Focusing on the Pediatric Population. *American Journal of Infection Control* **2013**, *41*, S130-S131, doi:10.1016/j.ajic.2013.03.258.
158. Chaudhary, M.; Bilal, M.; Du, W.; Chu, R.; McGrath, E.; Rajpurkar, M. Abstracts of the American Society of Pediatric Hematology/Oncology (ASPHO) 26th Annual Meeting. Miami, Florida, USA. April 24-27, 2013. *Pediatr Blood Cancer* **2013**, *60 Suppl 2*, S1-105, doi:10.1002/pbc.24509.
159. Chaudhary, M.; Bilal, M.F.; Du, W.; Chu, R.; Rajpurkar, M.; McGrath, E.J. The impact of ethanol lock therapy on length of stay and catheter salvage in pediatric catheter-associated bloodstream infection. *Clin Pediatr (Phila)* **2014**, *53*, 1069-1076, doi:10.1177/0009922814533591.
160. Chong, C.Y.; Ong, R.; Tan, N.; Thoon, K.C.; Seah, V. Taurolidine-citrate Lock Solution for the Prevention of Central-line Associated Bloodstream Infection. *American Journal of Infection Control* **2014**, *42*, S132-S133, doi:10.1016/j.ajic.2014.03.283.
161. Chu, H.P.; Brind, J.; Tomar, R.; Hill, S. Significant reduction in central venous catheter-related bloodstream infections in children on HPN after starting treatment with taurolidine line lock. *J Pediatr Gastroenterol Nutr* **2012**, *55*, 403-407, doi:10.1097/MPG.0b013e31825bb0ae.
162. Cuntz, D.; Michaud, L.; Guimber, D.; Husson, M.O.; Gottrand, F.; Turck, D. Local antibiotic lock for the treatment of infections related to central catheters in parenteral nutrition in children. *JPEN J Parenter Enteral Nutr* **2002**, *26*, 104-108, doi:10.1177/0148607102026002104.
163. Davidge, K.; Mroczka, E.; Ivy, C.; Nagel, J. 1680. Characterization of Ethanol Lock Use and Line Infections in Pediatric Patients with Intestinal Failure Requiring Central Venous Access. *Open Forum Infectious Diseases* **2023**, *10*, doi:10.1093/ofid/ofad500.1513.
164. de Frutos Porras, E.; Cobo-Vazquez, E.; Hernanz Lobo, A.; Santos Sebastian, M.D.M.; Perez Fernandez, E.; Garrido Colino, C.; Cela, E.; Navarro Gomez, M.L. A Local Experience of Antibiotic Lock Therapy as an Adjunctive Treatment for Central Venous Catheter-Related Bloodstream Infections in Pediatric Oncology and Hematology Patients. *Children (Basel)* **2024**, *11*, doi:10.3390/children11080983.
165. Demirok, A.; Illy, D.H.C.; Nagelkerke, S.Q.; Lagerweij, M.F.; Benninga, M.A.; Tabbers, M.M. Catheter salvage or removal in catheter-related bloodstream infections with Staphylococcus aureus in children with chronic intestinal failure receiving home parenteral nutrition and the use of prophylactic taurolidine catheter lock solution: A descriptive cohort study. *JPEN J Parenter Enteral Nutr* **2024**, *48*, 486-494, doi:10.1002/jpen.2630.
166. Goulet, O. 260.6: PEDIATRIC HOME PARENTERAL NUTRITION (HPN) IN FRANCE: a national survey On the behalf of the French Pediatric HPN network. *Transplantation* **2019**, *103*, S10-S10, doi:10.1097/01.tp.0000575456.12137.bf.

167. Hess, R.A.; Welch, K.B.; Brown, P.I.; Teitelbaum, D.H. Survival outcomes of pediatric intestinal failure patients: analysis of factors contributing to improved survival over the past two decades. *J Surg Res* **2011**, *170*, 27-31, doi:10.1016/j.jss.2011.03.037.
168. Josyabhatla, R.; Naik, M.; Liu, Y.; Speer, A.L.; Imseis, E.M. Sodium Bicarbonate Locks May Be a Safe and Effective Alternative in Pediatric Intestinal Failure: A Pilot Study. *J Pediatr Gastroenterol Nutr* **2022**, *75*, 304-307, doi:10.1097/MPG.0000000000003506.
169. Liang, H.; Zhang, L.; Guo, X.; Sun, L. Vancomycin-lock therapy for prevention of catheter-related bloodstream infection in very low body weight infants. **2020**, doi:10.21203/rs.3.rs-35490/v5.
170. McGhee, W.; Michaels, M.G.; Martin, J.M.; Mazariegos, G.V.; Green, M. Antifungal Lock Therapy with Liposomal Amphotericin B: A Prospective Trial. *J Pediatric Infect Dis Soc* **2016**, *5*, 80-84, doi:10.1093/jpids/piu083.
171. McNevin, K.; Rosete, B.; Fukasawa, S.; Wang, X.; Javid, P.; Wendel, D. ETHANOL COMPARED TO SODIUM BICARBONATE LINE LOCKS IN PEDIATRIC INTESTINAL FAILURE PATIENTS. *Journal of Pediatric Gastroenterology and Nutrition* **2023**, *77*, S317-S318.
172. Megged, O.; Shalit, I.; Yaniv, I.; Fisher, S.; Livni, G.; Levy, I. Outcome of antibiotic lock technique for persistent central venous catheter-associated coagulase-negative Staphylococcus bacteremia in children. *Eur J Clin Microbiol Infect Dis* **2010**, *29*, 157-161, doi:10.1007/s10096-009-0831-4.
173. Mohandas, A.; Jalil, M.; Isava-Quintero, A.; Duro, D. NASPGHAN Annual Meeting Abstracts. *Journal of Pediatric Gastroenterology and Nutrition* **2019**, *69*, doi:10.1097/mpg.0000000000002518.
174. Mokha, J.; Davidovics, Z.H.; Emerick, K.; Samela, K. Ethanol lock therapy in central venous catheters of children with intestinal failure is associated with lower rates of infection but higher rates of mechanical problems and catheter replacements. *Gastroenterology* **2015**, *148*, S630.
175. Mokha, J.S.; Davidovics, Z.H.; Samela, K.; Emerick, K. Effects of Ethanol Lock Therapy on Central Line Infections and Mechanical Problems in Children With Intestinal Failure. *JPEN J Parenter Enteral Nutr* **2017**, *41*, 625-631, doi:10.1177/0148607115625057.
176. Mouw, E.; Chessman, K.; Lesher, A.; Tagge, E. Use of an ethanol lock to prevent catheter-related infections in children with short bowel syndrome. *J Pediatr Surg* **2008**, *43*, 1025-1029, doi:10.1016/j.jpedsurg.2008.02.026.
177. Okur Acar, S.; Tahta, N.; Boncuoglu, E.; Odaman Al, I.; Kiymet, E.; Gozmen, S.; Demirag, B.; Karapinar, T.H.; Oymak, Y.; Vergin, C.; et al. Efficacy of Teicoplanin Lock Therapy in the Treatment of Port-related Coagulase-negative Staphylococci Bacteremia in Pediatric Oncology Patients. *J Pediatr Hematol Oncol* **2023**, *45*, e17-e20, doi:10.1097/MPH.0000000000002502.
178. Onland, W.; Shin, C.E.; Fustar, S.; Rushing, T.; Wong, W.Y. Ethanol-lock technique for persistent bacteremia of long-term intravascular devices in pediatric patients. *Arch Pediatr Adolesc Med* **2006**, *160*, 1049-1053, doi:10.1001/archpedi.160.10.1049.
179. Ozen, S.; Kanik Yuksek, S.; Dinc, B.; Uckardes, F.; Konca, H.K.; Erat, T.; Guney, A.Y.; Gunes, O.; Coskun, Z.N.; Gulhan, B.; et al. Catheter-related Infections in Pediatric Patients Due to a Rare Pathogen: Herbaspirillum huttiense. *Pediatr Infect Dis J* **2024**, *43*, e231-e234, doi:10.1097/INF.0000000000004350.
180. Pace, D.; Mack, S.; Berman, L.; Reichard, K.; Martin, A. Harry M. Vars Award Candidate Abstracts. *Journal of Parenteral and Enteral Nutrition* **2023**, *47*, S59-S60, doi:10.1002/jpen.2488.
181. Pitts, S.; Bergamo, D.; Cartaya, C.; Gore, B. Efficacy in the Reduction of Central Line-Associated Bloodstream Infection in a Patient With Intestinal Failure: An Ethanol Lock Pediatric Case Study. *Journal of the Association for Vascular Access* **2014**, *19*, 217-220, doi:10.1016/j.java.2014.07.003.
182. Qi, K.; Wong, E.; Peebles, E.; Atkison, P. Adjunctive Ethanol-Lock Therapy in Paediatric Patients with Catheter-Related Bloodstream Infection. *Canadian Journal of Hospital Pharmacy* **2016**, *60*, 87.

183. Quirt, J.; Belza, C.; Pai, N.; Clause, R.F.; Markovic, F.; Wong-Sterling, S.; Avitzur, Y.; Wales, P.W. Reduction of Central Line-Associated Bloodstream Infections and Line Occlusions in Pediatric Intestinal Failure Patients Receiving Long-Term Parenteral Nutrition Using an Alternative Locking Solution, 4% Tetrasodium Ethylenediaminetetraacetic Acid. *JPEN J Parenter Enteral Nutr* **2021**, *45*, 1286-1292, doi:10.1002/jpen.1989.
184. Restrepo, D.; Laconi, N.S.; Alcantar, N.A.; West, L.A.; Buttice, A.L.; Patel, S.; Kayton, M.L. Inhibition of heparin precipitation, bacterial growth, and fungal growth with a combined isopropanol-ethanol locking solution for vascular access devices. *J Pediatr Surg* **2015**, *50*, 472-477, doi:10.1016/j.jpedsurg.2014.07.003.
185. Robbins Tighe, S.L. Clinical Application of Prophylactic Ethanol Lock Therapy in Pediatric Patients With Intestinal Failure. *Gastroenterol Nurs* **2016**, *39*, 376-384, doi:10.1097/SGA.0000000000000180.
186. Savarese, I.; Yazami, S.; De Rose, D.U.; Carkeek, K.; Campi, F.; Auriti, C.; Danhaive, O.; Piersigilli, F. Use of 2% taurolidine lock solution for treatment and prevention of catheter-related bloodstream infections in neonates: a feasibility study. *J Hosp Infect* **2024**, *143*, 76-81, doi:10.1016/j.jhin.2023.11.003.
187. Shenep, L.E.; Shenep, M.A.; Cheatham, W.; Hoffman, J.M.; Hale, A.; Williams, B.F.; Perkins, R.; Hewitt, C.B.; Hayden, R.T.; Shenep, J.L. Efficacy of intravascular catheter lock solutions containing preservatives in the prevention of microbial colonization. *J Hosp Infect* **2011**, *79*, 317-322, doi:10.1016/j.jhin.2011.07.010.
188. Signorino, C.; Fusco, E.; Galli, L.; Chiappini, E. Effectiveness of Antimicrobial Lock Therapy for the Treatment of Catheter-Related and Central-Line-Associated Bloodstream Infections in Children: A Single Center Retrospective Study. *Antibiotics (Basel)* **2023**, *12*, doi:10.3390/antibiotics12050800.
189. Stefanidis, C.J. Prevention of catheter-related bacteremia in children on hemodialysis: time for action. *Pediatr Nephrol* **2009**, *24*, 2087-2095, doi:10.1007/s00467-009-1254-2.
190. Sun, Y.; Wan, G.; Liang, L. Taurolidine lock solution for catheter-related bloodstream infections in pediatric patients: A meta-analysis. *PLoS One* **2020**, *15*, e0231110, doi:10.1371/journal.pone.0231110.
191. Valentine, K.M. Ethanol lock therapy for catheter-associated blood stream infections in a pediatric intensive care unit. *Pediatr Crit Care Med* **2011**, *12*, e292-296, doi:10.1097/PCC.0b013e318219267c.
192. Walker, L.W.; Visweswaran, S.; Nowalk, A.J. Outcomes in Pediatric Central Line-associated Bloodstream Infections Treated With Antimicrobial Locks: A 14-Year Retrospective Analysis. *Pediatr Infect Dis J* **2023**, *42*, 473-478, doi:10.1097/INF.0000000000003885.
193. Weber, M.D.; Woods-Hill, C.; Resendiz, K.; Nelson, E.; Ryan, M.; Brennan, L.; Srinivasan, A.; Conlon, T. Safety and Efficacy of Ethanol for Catheter Salvage and Central Line-Associated Bloodstream Infection Prophylaxis in Polyurethane Catheters in the PICU. *Pediatr Crit Care Med* **2024**, *25*, e232-e238, doi:10.1097/PCC.0000000000003454.
194. Wolf, J.; Allison, K.J.; Tang, L.; Sun, Y.; Hayden, R.T.; Flynn, P.M. No evidence of benefit from antibiotic lock therapy in pediatric oncology patients with central line-related bloodstream infection: results of a retrospective matched cohort study and review of the literature. *Pediatr Blood Cancer* **2014**, *61*, 1811-1815, doi:10.1002/pbc.25101.
195. Xiao, X.L.; Yang, Q.X.; Niu, H.Z.; Li, L.J.; Xie, Z.J. A Retrospective Study of the Use of Antibiotic Lock Therapy and Cluster Nursing Management in Infections in Children with Short Bowel Syndrome or Solid Abdominal Tumours Treated with Totally Implantable Venous Access Ports. *J Multidiscip Healthc* **2023**, *16*, 431-438, doi:10.2147/JMDH.S397737.
196. Onder, A.M.; Billings, A.A.; Chandar, J.; Nield, L.; Francoeur, D.; Simon, N.; Abitbol, C.; Zilleruelo, G. Antibiotic lock solutions allow less systemic antibiotic exposure and less catheter malfunction without adversely affecting antimicrobial resistance patterns. *Hemodial Int* **2013**, *17*, 75-85, doi:10.1111/j.1542-4758.2012.00717.x.

197. Onder, A.M.; Chandar, J.; Billings, A.A.; Simon, N.; Diaz, R.; Francoeur, D.; Abitbol, C.; Zilleruelo, G. Comparison of early versus late use of antibiotic locks in the treatment of catheter-related bacteremia. *Clin J Am Soc Nephrol* **2008**, *3*, 1048-1056, doi:10.2215/CJN.04931107.
198. Onder, A.M.; Chandar, J.; Billings, A.; Simon, N.; Gonzalez, J.; Francoeur, D.; Abitbol, C.; Zilleruelo, G. Prophylaxis of catheter-related bacteremia using tissue plasminogen activator-tobramycin locks. *Pediatr Nephrol* **2009**, *24*, 2233-2243, doi:10.1007/s00467-009-1235-5.
199. Pérez, M.J.; Barrio, J.M.; Rincón, C.; Hortal, J.; Martín-Rabadán, P.; Pernia, S.; Bouza, E. Poster Sessions. *Clinical Microbiology and Infection* **2012**, *18*, 114-715, doi:10.1111/j.1469-0691.2012.03802.x.
200. Ranganath, N.; Dumais, M.; Nair, V.; Tabaja, H.; Stevens, R.W.W.; O'Horo, J.C.; Shah, A. 2432. Antibiotic Lock Therapy as a Line Salvage Strategy for Catheter-Related Bloodstream Infections: A Retrospective analysis. *Open Forum Infectious Diseases* **2023**, *10*, doi:10.1093/ofid/ofad500.2051.
201. Rojas-Moreno, C.A.; Spiegel, D.; Yalamanchili, V.; Kuo, E.; Quinones, H.; Sreeramoju, P.V.; Luby, J.P. Catheter-Related Bloodstream Infections in Patients on Emergent Hemodialysis. *Infect Control Hosp Epidemiol* **2016**, *37*, 301-305, doi:10.1017/ice.2015.293.
202. Krishna, A.; Navalkele, B.; Chandramohan, S.; Chopra, T. 2117. Catheter-related Bacteremia in Hemodialysis Patients on Antibiotic Lock Therapy: Are Antibiotic Locks Ineffective? *Open Forum Infectious Diseases* **2018**, *5*, S621-S621, doi:10.1093/ofid/ofy210.1773.
203. Blanco-Di Matteo, A.; Garcia-Fernandez, N.; Aguinaga Perez, A.; Carmona-Torre, F.; Oteiza, A.C.; Leiva, J.; Del Pozo, J.L. Pre-Emptive Antimicrobial Locks Decrease Long-Term Catheter-Related Bloodstream Infections in Hemodialysis Patients. *Antibiotics (Basel)* **2022**, *11*, doi:10.3390/antibiotics11121692.
204. Chow, K.M.; Poon, Y.L.; Lam, M.P.; Poon, K.L.; Szeto, C.C.; Li, P.K. Antibiotic lock solutions for the prevention of catheter-related bacteraemia in haemodialysis patients. *Hong Kong Med J* **2010**, *16*, 269-274.
205. Delgado, O.I.; Soto-Vargas, J.; López, H.R.; Reyes, J.F.T.; Lopez, M.A.S.; Garcia, O.C.M.; Pazarin-Villaseor, L. An outbreak of catheter-related bacteremia on hemodialysis patients caused by serratia marcescens. *Journal of the American Society of Nephrology* **2017**, *28*, 328-329.
206. Gang, S.; Konnur, A.; Rajapurkar, M.; Hegde, U.; Patel, H.; Patel, J.; Shete, N. Role of short-dwell daily ethanol-lock therapy in the management of hemodialysis tunneled cuffed catheter-related bloodstream infection. *J Vasc Access* **2024**, *25*, 1100-1107, doi:10.1177/11297298221149477.
207. Hussein, W.F.; Gomez, N.; Sun, S.J.; Yu, J.; Yang, F.; Ajuria, M.; Abra, G.E.; Schiller, B. Use of a gentamicin-citrate lock leads to lower catheter-related bloodstream infection rates and reduced cost of care in hemodialysis patients. *Hemodial Int* **2021**, *25*, 20-28, doi:10.1111/hdi.12880.
208. Joshi, A.J.; Hart, P.D. Antibiotic catheter locks in the treatment of tunneled hemodialysis catheter-related blood stream infection. *Semin Dial* **2013**, *26*, 223-226, doi:10.1111/j.1525-139X.2012.01115.x.
209. Landry, D.L.; Braden, G.L.; Gobeille, S.L.; Haessler, S.D.; Vaidya, C.K.; Sweet, S.J. Emergence of gentamicin-resistant bacteremia in hemodialysis patients receiving gentamicin lock catheter prophylaxis. *Clin J Am Soc Nephrol* **2010**, *5*, 1799-1804, doi:10.2215/CJN.01270210.
210. Lau, S.; Kam, L.W.; Lamarche, J.; Peguero, A.M. The Effectiveness of Antibiotic-Lock Therapy for the Prevention of Catheter-Related Bacteremia in Patients on Chronic Hemodialysis: Case Controlled Study in a Veteran Population. *Nephrology Research & Reviews* **2014**, *1*, 18-21, doi:10.4081/nr.2009.e5.
211. Merino, J.L.; Bouarich, H.; Pita, M.J.; Martinez, P.; Bueno, B.; Caldes, S.; Corchete, E.; Jaldo, M.T.; Espejo, B.; Paraiso, V. Serratia marcescens bacteraemia outbreak in haemodialysis patients with tunnelled catheters due to colonisation of antiseptic solution. Experience at 4 hospitals. *Nefrologia* **2016**, *36*, 667-673, doi:10.1016/j.nefro.2016.05.009.

212. Neusser, M.A.; Bobe, I.; Hammermeister, A.; Wittmann, U. A 2% taurolidine catheter lock solution prevents catheter-related bloodstream infection (CRBSI) and catheter dysfunction in hemodialysis patients. *Br J Nurs* **2021**, *30*, S24-S32, doi:10.12968/bjon.2021.30.14.S24.
213. Rijnders, B.J.; Slobbe, L. Abstracts of the 20th ECCMID (European Congress of Clinical Microbiology and Infectious Diseases). Vienna, Austria. April 10-13, 2010. *Clin Microbiol Infect* **2010**, *16 Suppl 2*, S1-720, doi:10.1111/j.1469-0691.2010.03238.x.
214. Saxena, A.K.; Panhotra, B.R.; Al-hafiz, A.A.; Sundaram, D.S.; Abu-Oyun, B.; Al Mulhim, K. Cefotaxime-heparin lock prophylaxis against hemodialysis catheter-related sepsis among Staphylococcus aureus nasal carriers. *Saudi J Kidney Dis Transpl* **2012**, *23*, 743-754, doi:10.4103/1319-2442.98154.
215. Saxena, A.K.; Panhotra, B.R.; Sundaram, D.S.; Al-Hafiz, A.; Naguib, M.; Venkateshappa, C.K.; Abu-Oun, B.A.; Hussain, S.M.; Al-Ghamdi, A.A. Tunneled catheters' outcome optimization among diabetics on dialysis through antibiotic-lock placement. *Kidney Int* **2006**, *70*, 1629-1635, doi:10.1038/sj.ki.5001776.
216. Solomon, L.R.; Cheesbrough, J.S.; Bhargava, R.; Mitsides, N.; Heap, M.; Green, G.; Diggle, P. Observational study of need for thrombolytic therapy and incidence of bacteremia using taurolidine-citrate-heparin, taurolidine-citrate and heparin catheter locks in patients treated with hemodialysis. *Semin Dial* **2012**, *25*, 233-238, doi:10.1111/j.1525-139X.2011.00951.x.
217. Winnicki, W.; Herkner, H.; Lorenz, M.; Handisurya, A.; Kikic, Z.; Bielesz, B.; Schairer, B.; Reiter, T.; Eskandary, F.; Sunder-Plassmann, G.; et al. Taurolidine-based catheter lock regimen significantly reduces overall costs, infection, and dysfunction rates of tunneled hemodialysis catheters. *Kidney Int* **2018**, *93*, 753-760, doi:10.1016/j.kint.2017.06.026.
218. Yen, H.W.; Yang, W.C.; Tarng, D.C.; Yang, C.Y.; Chuang, C.L.; Huang, L.J.; Lin, P.Y.; Wang, C.C.; Li, S.Y. Daptomycin antibiotic lock therapy for hemodialysis patients with Gram-positive bloodstream infections following use of tunneled, cuffed hemodialysis catheters: retrospective single center analysis. *Hemodial Int* **2016**, *20*, 315-320, doi:10.1111/hdi.12378.
219. Zwiech, R.; Adelt, M.; Chrul, S. A Taurolidine-Citrate-Heparin Lock Solution Effectively Eradicates Pathogens From the Catheter Biofilm in Hemodialysis Patients. *Am J Ther* **2016**, *23*, e363-368, doi:10.1097/MJT.0b013e31828d4610.
220. Gundogan, K.; Dave, N.J.; Griffith, D.P.; Zhao, V.M.; McNally, T.A.; Easley, K.A.; Haack, C.I.; Galloway, J.R.; Ziegler, T.R. Ethanol Lock Therapy Markedly Reduces Catheter-Related Blood Stream Infections in Adults Requiring Home Parenteral Nutrition: A Retrospective Study From a Tertiary Medical Center. *JPEN J Parenter Enteral Nutr* **2020**, *44*, 661-667, doi:10.1002/jpen.1698.
221. Sanchez-Munoz, A.; Aguado, J.M.; Lopez-Martin, A.; Lopez-Medrano, F.; Lumbreras, C.; Rodriguez, F.J.; Colomer, R.; Cortes-Funes, H. Usefulness of antibiotic-lock technique in management of oncology patients with uncomplicated bacteremia related to tunneled catheters. *Eur J Clin Microbiol Infect Dis* **2005**, *24*, 291-293, doi:10.1007/s10096-005-1291-0.
222. Dannenberg, C.; Bierbach, U.; Rothe, A.; Beer, J.; Korholz, D. Ethanol-lock technique in the treatment of bloodstream infections in pediatric oncology patients with broviac catheter. *J Pediatr Hematol Oncol* **2003**, *25*, 616-621, doi:10.1097/00043426-200308000-00006.
223. Kawano, T.; Kaji, T.; Onishi, S.; Yamada, K.; Yamada, W.; Nakame, K.; Mukai, M.; Ieiri, S. Efficacy of ethanol locks to reduce the incidence of catheter-related bloodstream infections for home parenteral nutrition pediatric patients: comparison of therapeutic treatment with prophylactic treatment. *Pediatr Surg Int* **2016**, *32*, 863-867, doi:10.1007/s00383-016-3933-y.
224. Broom, J. P601 Ethanol lock therapy: preliminary trial results and future research agenda. *International Journal of Antimicrobial Agents* **2007**, *29*, S541-S542, doi:10.1016/s0924-8579(07)70444-2.
225. Rinke, M.L.; Heo, M.; Saiman, L.; Bundy, D.G.; Rosenberg, R.E.; DeLaMora, P.; Rabin, B.; Zachariah, P.; Mirhaji, P.; Ford, W.J.H.; et al. Pediatric Ambulatory Central Line-Associated Bloodstream Infections. *Pediatrics* **2021**, *147*, doi:10.1542/peds.2020-0524.

226. Alonso, B.; Fernandez-Cruz, A.; Diaz, M.; Sanchez-Carrillo, C.; Martin-Rabadan, P.; Bouza, E.; Munoz, P.; Guembe, M. Can vancomycin lock therapy extend the retention time of infected long-term catheters? *APMIS* **2020**, *128*, 433-439, doi:10.1111/apm.13033.
227. Ashkenazi-Hoffnung, L.; Shecter, N.; De-Vries, I.; Levy, I.; Scheuerman, O.; Yarden-Bilavsky, H.; Bernfeld, Y.; Mor, M. Factors predicting efficacy of ethanol lock therapy as catheter salvage strategy for pediatric catheter-related infections. *Pediatr Blood Cancer* **2021**, *68*, e28856, doi:10.1002/pbc.28856.
228. Aysert-Yildiz, P.; Kalkanci, A.; Erdogan, M.; Ozger, H.S.; Ozturk, A.; Guzel-Tunccan, O.; Dizbay, M.; Caglar, K. The effectiveness of antimicrobial photodynamic therapy on catheter infection model. *J Photochem Photobiol B* **2024**, *260*, 113026, doi:10.1016/j.jphotobiol.2024.113026.
229. Begum, N.A.S.; Kashem, T.S.; Arefin, S.U.; Rashid, H.U. Sat-228 Comparing Antibiotic Lock Therapy with Systemic Antibiotics to Prevent Central Line Associated Blood Stream Infection (Clabsi). *Kidney International Reports* **2020**, *5*, S97-S98, doi:10.1016/j.ekir.2020.02.243.
230. Broom, J. P601 Ethanol lock therapy: preliminary trial results and future research agenda. *International Journal of Antimicrobial Agents* **2007**, *29*, doi:10.1016/s0924-8579(07)70444-2.
231. Cullis, P.S.; McKee, R.F. Taurolidine lock - experience from the West of Scotland. *Clin Nutr* **2011**, *30*, 399-400; author reply 401, doi:10.1016/j.clnu.2010.12.008.
232. Del Pozo, J.L.; Rodil, R.; Aguinaga, A.; Yuste, J.R.; Bustos, C.; Montero, A.; Espinosa, G.; Garcia-Fernandez, N. Daptomycin lock therapy for grampositive long-term catheter-related bloodstream infections. *Int J Clin Pract* **2012**, *66*, 305-308, doi:10.1111/j.1742-1241.2011.02830.x.
233. Epperson, E.L. Efficacy of 0.9% sodium chloride injection with and without heparin for maintaining indwelling intermittent injection sites. *Clin Pharm* **1984**, *3*, 626-629.
234. Fligor, S.C.; Hirsch, T.I.; Tsikis, S.T.; Joiner, M.M.; Mitchell, P.D.; Carbeau, S.; McClelland, J.; Carey, A.; Gura, K.M.; Puder, M. Ethanol lock therapy increases mechanical catheter complications in a pediatric intestinal failure population: A retrospective cohort study. *JPEN J Parenter Enteral Nutr* **2023**, *47*, 662-669, doi:10.1002/jpen.2509.
235. Freire, M.P.; Pierrotti, L.C.; Zerati, A.E.; Benites, L.; da Motta-Leal Filho, J.M.; Ibrahim, K.Y.; Araujo, P.H.; Abdala, E. Role of Lock Therapy for Long-Term Catheter-Related Infections by Multidrug-Resistant Bacteria. *Antimicrob Agents Chemother* **2018**, *62*, doi:10.1128/AAC.00569-18.
236. Ghannoum, M.A.; Isham, N.; Jacobs, M.R. Antimicrobial activity of B-Lock against bacterial and Candida spp. causing catheter-related bloodstream infections. *Antimicrob Agents Chemother* **2011**, *55*, 4430-4431, doi:10.1128/AAC.00677-11.
237. Ince, E.; Oguzkurt, P.; Temiz, A.; Ezer, S.S.; Gezer, H.O.; Yazici, N.; Hicsonmez, A. Complications of total implantable access ports and efficacy of Taurolidine-citrate lock solution against catheter-related infections. *Afr J Paediatr Surg* **2014**, *11*, 138-142, doi:10.4103/0189-6725.132806.
238. Kaufman, L.; Kelly, D.; Oxentenko, A. Prophylactic Antibiotic Lock Use Significantly Decreases the Incidence of Bloodstream Infections in Patients Requiring Home Parenteral Nutrition: The Mayo Experience: Presidential Poster: 295. *Official journal of the American College of Gastroenterology | ACG* **2012**, *107*, S127.
239. Krumpelmann, U.; Boseila, A.; Lohnert, M.; Kaup, O.; Clarenbach, J.J.; Gorner, M. An analysis of totally implantable central venous port system infections in an urban tertiary referral center. *J Chemother* **2021**, *33*, 228-237, doi:10.1080/1120009X.2020.1829327.
240. Lawinski, M.; Majewska, K.; Gradowski, L.; Foltyn, I.; Singer, P. A comparison of two methods of treatment for catheter-related bloodstream infections in patients on home parenteral nutrition. *Clin Nutr* **2015**, *34*, 918-922, doi:10.1016/j.clnu.2014.09.013.
241. Marschall, J.; Mermel, L.A.; Fakih, M.; Hadaway, L.; Kallen, A.; O'Grady, N.P.; Pettis, A.M.; Rupp, M.E.; Sandora, T.; Maragakis, L.L.; et al. Strategies to prevent central line-associated bloodstream infections in acute care hospitals: 2014 update. *Infect Control Hosp Epidemiol* **2014**, *35*, 753-771, doi:10.1086/676533.

242. Meckmongkol, T.T.; Costanzo, C.; Ciullo, S.; Prasad, R.; Arthur, L.G. Hidden morbidity of ethanol lock therapy. *Pediatr Surg Int* **2018**, *34*, 71-74, doi:10.1007/s00383-017-4168-2.
243. Pomplun, M.; Johnson, J.J.; Johnston, S.; Kolesar, J.M. Stability of a heparin-free 50% ethanol lock solution for central venous catheters. *J Oncol Pharm Pract* **2007**, *13*, 33-37, doi:10.1177/1078155207077598.
244. Rajpurkar, M.; McGrath, E.; Joyce, J.; Boldt-MacDonald, K.; Chitlur, M.; Lusher, J. Therapeutic and prophylactic ethanol lock therapy in patients with bleeding disorders. *Haemophilia* **2014**, *20*, 52-57, doi:10.1111/hae.12241.
245. Ranch-Lundin, M.; Schedin, A.; Bjorkhem-Bergman, L. Equal effect of vancomycin lock with or without heparin in treatment of central venous catheter related blood stream infections - an observational study in palliative home care. *Infect Dis (Lond)* **2021**, *53*, 719-723, doi:10.1080/23744235.2021.1922752.
246. Sodemann, K.; Polaschegg, H.D.; Feldmer, B. Two years' experience with Dialock and CLS (a new antimicrobial lock solution). *Blood Purif* **2001**, *19*, 251-254, doi:10.1159/000046950.
247. Soman, R.; Gupta, N.; Suthar, M.; Kothari, J.; Almeida, A.; Shetty, A.; Rodrigues, C. Antibiotic Lock Therapy in the Era of Gram-Negative Resistance. *J Assoc Physicians India* **2016**, *64*, 32-37.
248. Soriano, A.; Bregada, E.; Marques, J.M.; Ortega, M.; Bove, A.; Martinez, J.A.; Mensa, J. Decreasing gradient of antibiotic concentration in the lumen of catheters locked with vancomycin. *Eur J Clin Microbiol Infect Dis* **2007**, *26*, 659-661, doi:10.1007/s10096-007-0356-7.
249. Stein, R.A. Locking out the bugs: the first retrospective study on daptomycin lock therapy for catheter-related bloodstream infections. *Int J Clin Pract* **2012**, *66*, 231-233, doi:10.1111/j.1742-1241.2011.02881.x.
250. Tatarelli, P.; Parisini, A.; Del Bono, V.; Mikulska, M.; Viscoli, C. Efficacy of daptomycin lock therapy in the treatment of bloodstream infections related to long-term catheter. *Infection* **2015**, *43*, 107-109, doi:10.1007/s15010-014-0675-4.
251. Tejwani, R.; Parry, M.F. Antimicrobial Lock Therapy as an Adjunct to Management of Catheter-Related Bacteremia. *Infectious Diseases in Clinical Practice* **2011**, *19*, 256-261, doi:10.1097/IPC.0b013e31820a5443.
252. Vassallo, M.; Genillier, P.L.; Dunais, B.; Kaphan, R.; Saudes, L.; Duval, Y.; Rolland, F.; Jullien, V.; Weiss, N.; Blanchouin, E.; et al. Short-course daptomycin lock and systemic therapy for catheter-related bloodstream infections: a retrospective cohort study in cancer patients with surgically implanted devices. *J Chemother* **2017**, *29*, 232-237, doi:10.1080/1120009X.2017.1282335.
253. Viale, P.; Pagani, L.; Petrosillo, N.; Signorini, L.; Colombini, P.; Macri, G.; Cristini, F.; Gattuso, G.; Carosi, G.; Italian, H.; et al. Antibiotic lock-technique for the treatment of catheter-related bloodstream infections. *J Chemother* **2003**, *15*, 152-156, doi:10.1179/joc.2003.15.2.152.
254. Bueno, C.V.; Padillo, J.N.; Sacaluga, L.G.; Gil-Bermejo, J.M.; Torres, M.S.; Hidalgo, L.H.; Gil, A.G.; Navarro, M.G. 3PC-014 Stability study of gentamicin lock therapy with heparin or citrate as anticoagulant. *European Journal of Hospital Pharmacy* **2018-03-01**, *25*, doi:10.1136/ejhpharm-2018-eahpconf.66.
255. Zhao, T.; Liu, H.; Han, J. Ethanol lock is effective on reducing the incidence of tunneled catheter-related bloodstream infections in hemodialysis patients: a systematic review and meta-analysis. *Int Urol Nephrol* **2018**, *50*, 1643-1652, doi:10.1007/s11255-018-1855-4.
